# Performance of Microsoft Azure Kinect DK as a tool for estimating human body segment lengths

**DOI:** 10.1038/s41598-024-66798-0

**Published:** 2024-07-09

**Authors:** Shiou-An Wang, Ming-Hua Lu, Ai-Teng Lee, Chao-Yu Chen, Li-Wen Lee

**Affiliations:** 1 Department of Computer Science and Information Engineering, Hungkuo Delin University of Technology, New Taipei City, 236302 Taiwan; 2 Department of Diagnostic Radiology, Chang Gung Memorial Hospital, No. 8, Section West, Jiapu Road, Puzi City, Chiayi, 613016 Taiwan; 3 Department of Obstetrics and Gynecology, Chang Gung Memorial Hospital, Chiayi, 613016 Taiwan; 4 Graduate Institute of Clinical Medical Sciences, College of Medicine, Chang Gung University, Taoyuan, 33302 Taiwan; 5 School of Medicine, College of Medicine, Chang Gung University, Taoyuan, 33302 Taiwan

**Keywords:** Musculoskeletal system, Bone imaging, Whole body imaging

## Abstract

The Microsoft Kinect depth sensor, with its built-in software that automatically captures joint coordinates without markers, could be a potential tool for ergonomic studies. This study investigates the performance of Kinect in limb segment lengths using dual-energy X-ray absorptiometry (DXA) as a reference. Healthy children and adults (n = 76) were recruited for limb length measurements by Kinect and DXA. The results showed consistent ratios of arm, forearm, thigh, and leg lengths to height, which were 0.16, 0.14, 0.23, and 0.22 respectively, for both age groups and methods. Kinect exhibited perfect correlation among all limb lengths, indicating fixed proportions assumed by its algorithm. Comparing the two methods, there was a strong correlation (R = 0.850–0.985) and good to excellent agreement (ICC = 0.829–0.977), except for the right leg in adults, where agreement was slightly lower but still moderate (ICC = 0.712). The measurement bias between the methods ranged from − 1.455 to 0.536 cm. In conclusion, Kinect yields outcomes similar to DXA, indicating its potential utility as a tool for ergonomic studies. However, the built-in algorithm of Kinect assumes fixed limb proportions for individuals, which may not be ideal for studies focusing on investigating limb discrepancies or anatomical differences.

## Introduction

Anthropometry is an academic discipline that investigates the physical parameters related to body size and shapes, encompassing both static and dynamic aspects. This discipline offers critical insights into overall health status, nutritional well-being, and the growth and development of children. Anthropometric assessment can be acquired by clinical, photogrammetric or radiographic measurement. Clinical measurements mainly use anthropometric tools, including measuring tape, calipers, and weight scales^1^. Nevertheless, these methods are characterized by labor-intensive and time-consuming procedures and demand a high degree of expertise from professionals to yield precise results, making them impractical for extensive and frequent measurements in large-scale studies. Photogrammetric anthropometry, which uses photography to measure human body dimensions, shares similarities with clinical anthropometry in its reliance on manual marking of body segments, which can be labor-intensive^2^. Furthermore, it assesses body dimensions in 2D images, potentially leading to less accuracy. Radiographic anthropometric measurements use X-ray, CT or MRI images to calculate the distance between specific anatomical landmarks^3^. However, these methods are expensive and need to be done at a hospital and may involve the use of irradiation. Due to the limitations of current anthropometric methods, there is a need to develop automated, precise, and cost-effective tools in anthropometry to further public health research.

The Microsoft Kinect integrates a depth sensor, color video camera, microphone array and orientation sensor into an All-in-One device. The device provides marker-free, real-time 3D location of body joints during static poses and dynamic movements and may provide an automatic tool for anthropometrics. The first generation of Kinect sensor was announced in 2010 with the primary objective of enhancing human–computer interaction within the Xbox 360 gaming console. Subsequently, multiple generations of Kinect for Windows have been developed, thereby expanding the utilization of Kinect sensors into education, healthcare, retail, and transportation industries^4^. The Kinect for Windows version 1 (Kinect v1) was released in 2012 that employs a structured light technique for capturing depth information and is capable of tracking 20 human joints. In 2014, the Kinect for Windows version 2 (Kinect v2) was introduced, featuring time-of-flight technology for depth information and the capability to tract 25 human joints. In 2019, the latest generation of Kinect sensor, the Microsoft Azure Kinect DK (Azure Kinect), became commercially available, capable of tracking up to 32 body joints using time-of-flight technology. Currently, the Kinect sensor is used for 3D reconstruction to measure spine alignment^5,6^ and assess physical function by determining 3D joint positions^7^.

The Azure Kinect featured with more advanced depth sensors has improved spatial and temporal resolution over the previous generation of Kinect sensors. It is reported that the systematic spatial error of Azure Kinect is under 2 mm from a range of 1.0–2.0 m in front of the sensor^8,9^. However, its efficacy in the precise localization of body joints and quantification of body limb length remains to be substantiated. To fill in the research gap, a validation study of Azure Kinect for the body segment measurement was performed in this study. We chose dual-energy X-ray absorptiometry (DXA) as the reference method for Kinect measurement as DXA can provide direct visualization of skeleton and joint with only daily background radiation exposure whereas the rest of the anthropometric methods identify body joints indirectly by using body landmarks located closely to skeletal points^10^.

A whole-body DXA scan is primarily used for bone mineral density estimation and body composition analysis. It has also been used as a tool for limb length measurement after resizing and rescaling to actual dimensions^11,12^. We hypothesized that the DXA image would yield accurate anthropometric measurements. This study aimed to validate the use of Azure Kinect as a tool for limb length measurement in both healthy pediatric and adult population, utilizing DXA as the reference method.

## Material and methods

### Study design and ethical considerations

This prospective cross-sectional study was approved by the Institutional Review Board of the Chang Gung Medical Foundation (IRB No: 202002212A3). All procedures performed in studies involving human participants were in accordance with the ethical standards of the institutional committee and with the 1964 Helsinki Declaration and its later amendments or comparable ethical standards. Written informed consent was obtained from all participants, and from their parents or legal guardians of those under 18 years of age. The studies were conducted from January 2022 to September 2023.

### Participants

Eligible participants were healthy Taiwanese aged 6–60 years. Exclusion criteria were participants with pregnancy, known chronic disease, limb defect and pacemaker implant. Anthropometric and Kinect measures were performed by trained research assistants. DXA examinations were conducted by a certified DXA technician. All measurements were conducted at the Radiology Department of Chang Gung Memorial Hospital at Chiayi. Prior to the study, participants were instructed to fast for four hours and to empty their bladders.

### Anthropometric measure

Body height and weight were measured using a digital scale (Super-View, HW-3050, Taipei, Taiwan) with participants wearing no shoes and lightweight clothing. Weight measurements were recorded to the nearest 0.1 kg and height measurements were recorded to the nearest 0.1 cm. A single measurement was taken for each participant.

### Azure Kinect setup and data acquisition

**Figure 1:**
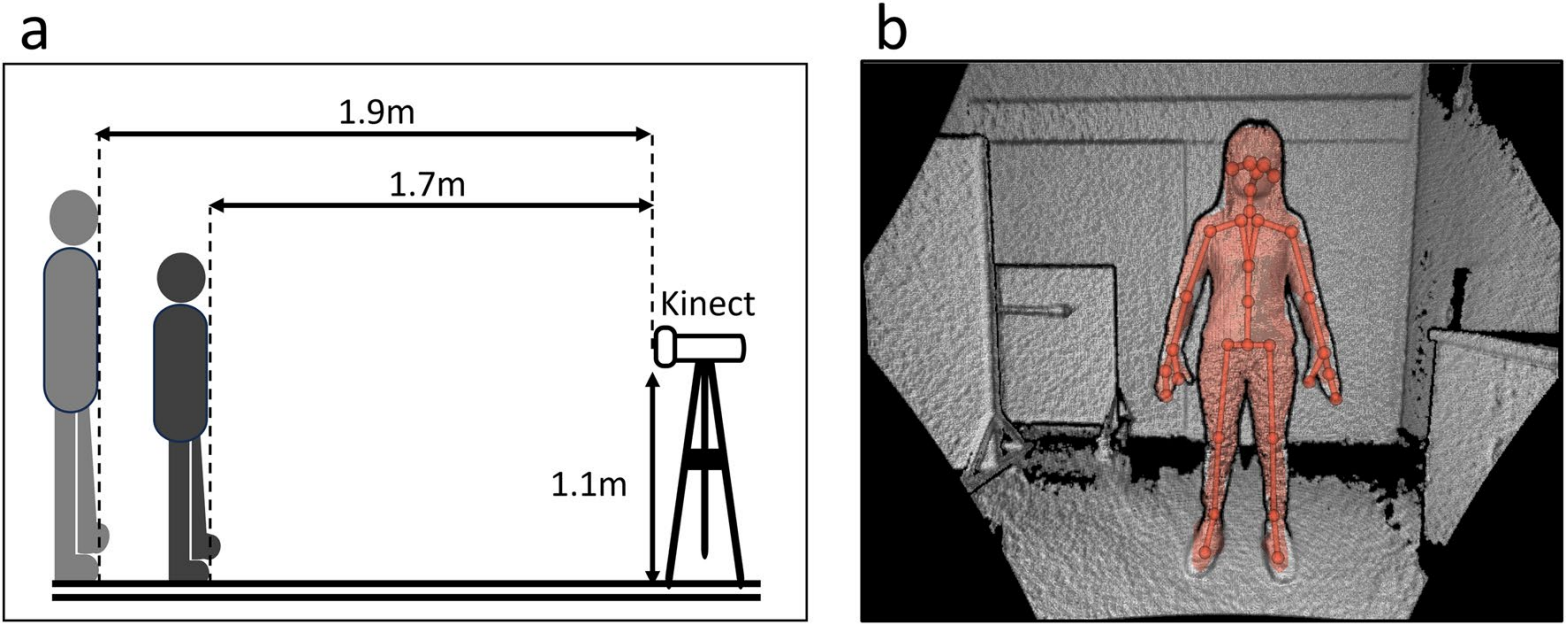
Experimental Setup for Azure Kinect Placement. (**a**) The Azure Kinect was placed on a tripod 1.1 m high, 1.7 m from the subject. If the subject’s height exceeded the sensor’s field of view, they stood 1.9 m in front of it. (**b**) Participant Skeletal Tracking Illustration. The image depicts a participant with skeletal tracking overlaid, showcasing the spatial tracking of 32 body points.

The Kinect measurements were conducted in a windowless examination room, primarily illuminated by uniform artificial lighting. The Azure Kinect DK (Microsoft Inc., Redmond, WA, USA) was positioned in front of the participant at a height of 1.1 m on a tripod, approximately 1.7 m away from the individual being recorded (Fig. 1A). For individuals whose height exceeded the field of view of the Kinect sensor, the tripod was positioned 1.9 m away from the recorded individual.

The Kinect sensor’s raw data was captured at a sampling rate of 30 Hz, utilizing the narrow field of view mode without binning, and possessing a resolution of 640 × 576. This was achieved through the integration of the Azure Kinect SDK v1.4.0 and Azure Kinect Body Tracking SDK v1.1.0, executed within the Visual Studio Code 2019 compiler environment using C/C# programming languages. This investigation was conducted using a Gigabyte laptop computer equipped with an 11th Generation Intel® Core™ i7-11800H Processor operating at boost clock speed of 4.6 GHz, and a NVIDIA GeForce RTX 3080 mobile graphics card, running on the Windows 10 operating system.

Participants were instructed to slowly move their limbs to allow the Kinect to capture the joints and then stand still with their legs apart and hands held away from their torso (Fig. 1B). Ten static depth images were captured and coordinated for measurements per participant after repositioning. The entire Kinect measurement process lasted approximately 15 min. The Azure Kinect Body Tracking SDK automatically provided 32 joint coordinates for the human body. This investigation focused on measuring eight major segments of the human limb, specifically the upper arm, forearm, thigh, and leg on both sides of the body.

### DXA setup and data acquisition

Whole body image was acquired using a fan-beam DXA system (Horizon W, Hologic, Inc.) equipped with Hologic Apex version 5.6. According to the manufacturer’s product specifications, a whole body DXA scan by the Hologic Horizon W scanner requires 272 s with 15 uSv radiation exposure and the scan length is 195.5 cm and scan width is 65.5 cm. Prior to image analysis, DXA images in JPEG format were downloaded, resized to their actual dimensions, and subsequently rescaled to the known scan length using Image J 1.54 (National Institutes of Health, Bethesda, MD, USA) by a trained research assistant. Then, a radiologist with more than 20 years of experience in DXA measured once from the final images, including the biomechanical lengths of the upper arm, forearm, thigh, and leg on both sides of the body^13,14^. The intraclass correlation coefficient (ICC) between a single rater’s three measurements on the same DXA images of the first 15 participants ranged from 0.960 to 0.996, indicating almost perfect agreement.

### Theory/calculation

To quantify the body segment lengths by Kinect, measurements were taken from the shoulder to the elbow for the upper arm, from the elbow to the wrist for the forearm, from the hip to the knee for the thigh, and from the knee to the ankle for the leg (Supplementary Table 1). The length of body segment was calculated by measuring distance between two joints, defined by their coordinates (x_1_, y_1_, z_1_) and (x_2_, y_2_, z_2_), using Eq. (1) as below:

1 $${\text{body}} \; {\text{segment}} \; {\text{length}}= \sqrt{{\left({x}_{2}-{x}_{1}\right)}^{2}+{\left({y}_{2}-{y}_{1}\right)}^{2}+{\left({z}_{2}-{z}_{1}\right)}^{2}}$$

**Figure 2:**
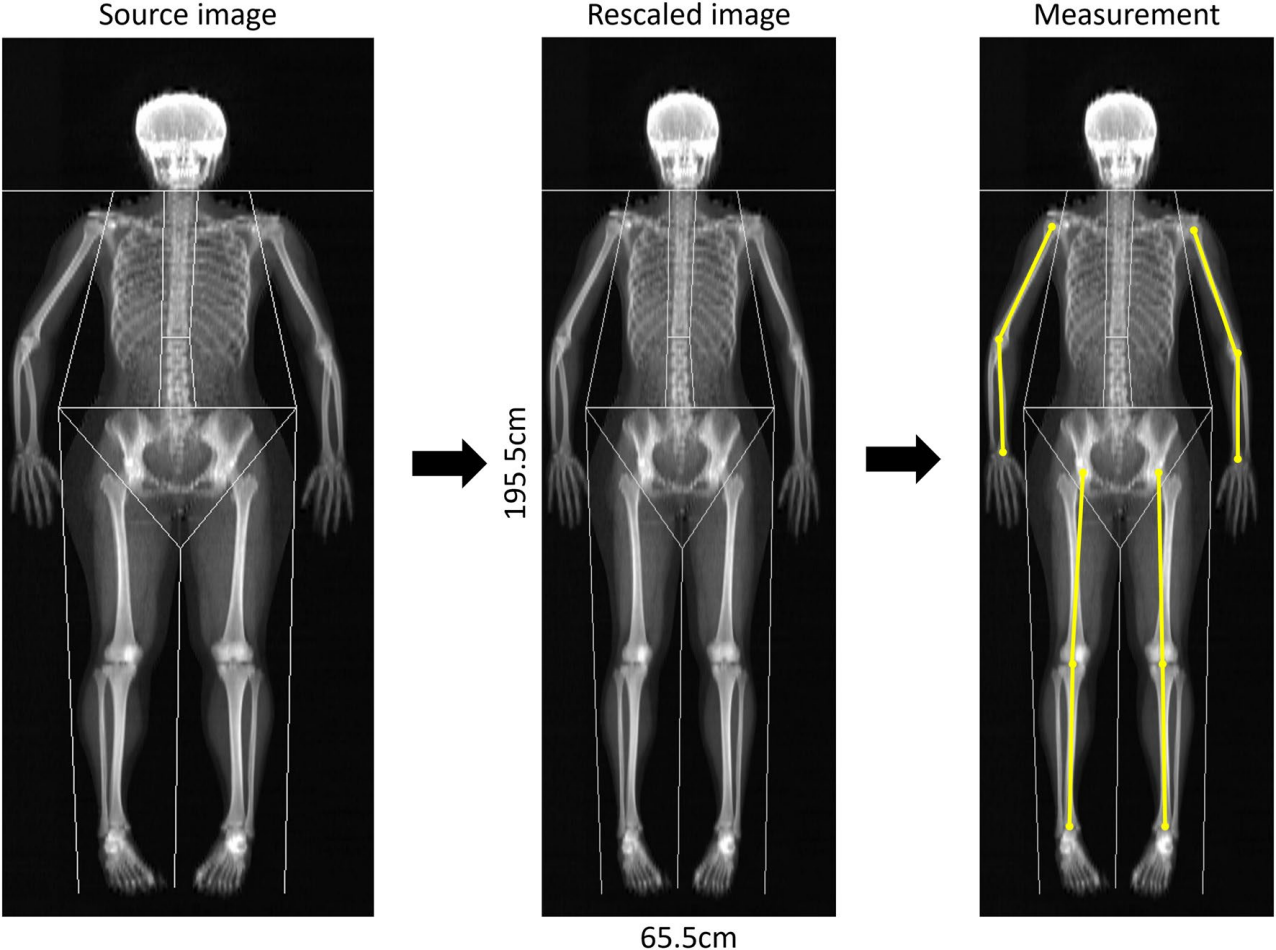
Imaging processing workflow for dual-energy X-ray absorptiometry image. A whole-body DXA scan is resized and rescaled to its actual dimensions of 195.5 cm in length and 65.5 cm in width using Image J. Subsequently, limb length measurements were obtained from the processed image.

To quantify the body segment lengths by DXA, measurements were taken from the center of the humeral head to the midpoint of the humeroradial joint for the upper arm, from the midpoint of the humeroradial joint to the midpoint of the radiocarpal joint for the forearm, from the femoral head center to the midpoint of the tibial condyles for the thigh, and from the midpoint of the tibial condyles to the mid-width of the talus for the leg (Fig. 2).

### Statistical analysis

Statistical analyses are performed using MedCalc for Windows (MedCalc Software, Ostend, Belgium). ICC for was employed to evaluate absolute agreement for the same rater among the initial 15 participants. Correlation Coefficient (R) and R-squared (R^2^) are used to represent the correlation between Kinect and DXA measurement methods. ICC for absolute agreement, Concordance Correlation Coefficient (CCC), and Bland–Altman Plot are used to demonstrate consistency between these two tools.

## Results

This study enrolled 22 children (11 boys and 11 girls) and 54 adults (25 males and 29 females) as participants. The average age of children was 13.18 years, while adults had an average age of 35.12 years. Participant characteristics are detailed in Table 1. Two children were found to be obese with BMI z-scores over 2, while seventeen adults were diagnosed with obesity due to their BMI exceeding 25 kg/m^2^. The average ratios of arm length to height, forearm length to height, thigh length to height and leg length to height were consistent at 0.16, 0.14, 0.23 and 0.22, respectively, across both children and adults, regardless of whether DXA or Azure Kinect methods were utilized.

**Table 1:** Subject characteristics.

Items	Children (n = 22)		Adults (n = 54)	
	Length (cm)	Proportions of height	Length (cm)	Proportions of height
Age (year)	13.18 (3.05)		35.12 (13.80)	
Height (cm)	156.5 (15.7)		167.2 (7.1)	
Weight (kg)	46.4 (13.3)		64.3 (13.4)	
BMI (kg/m^2^)	18.5 (3.2)		22.9 (3.7)	
DXA results				
Lt arm (cm)	25.1 (3.1)	0.160 (0.005)	26.8 (1.4)	0.160 (0.004)
Rt arm (cm)	25.2 (3.1)	0.161 (0.005)	26.9 (1.4)	0.161 (0.005)
Lt forearm (cm)	21.5 (2.4)	0.137 (0.005)	22.7 (1.4)	0.136 (0.005)
Rt forearm (cm)	21.4 (2.5)	0.137 (0.005)	22.9 (1.5)	0.137 (0.005)
Lt thigh (cm)	36.7 (4.4)	0.234 (0.008)	39.1 (2.3)	0.234 (0.007)
Rt thigh (cm)	36.7 (4.5)	0.234 (0.009)	39.1 (2.3)	0.234 (0.007)
Lt leg (cm)	34.4 (4.0)	0.220 (0.006)	36.1 (2.1)	0.216 (0.006)
Rt leg (cm)	34.4 (3.9)	0.219 (0.006)	35.9 (2.1)	0.215 (0.005)
Kinect results				
Lt arm (cm)	25.0 (2.6)	0.160 (0.002)	26.7 (1.4)	0.160 (0.003)
Rt arm (cm)	25.5 (2.7)	0.163 (0.002)	27.2 (1.4)	0.163 (0.003)
Lt forearm (cm)	21.0 (2.2)	0.134 (0.002)	22.4 (1.1)	0.134 (0.003)
Rt forearm (cm)	21.2 (2.2)	0.136 (0.002)	22.7 (1.2)	0.136 (0.003)
Lt thigh (cm)	36.2 (3.8)	0.231 (0.003)	38.7 (2.0)	0.232 (0.004)
Rt thigh (cm)	36.2 (3.8)	0.231 (0.003)	38.7 (2.0)	0.231 (0.004)
Lt leg (cm)	34.6 (3.6)	0.221 (0.003)	37.0 (1.9)	0.221 (0.004)
Rt leg (cm)	34.9 (3.7)	0.223 (0.003)	37.4 (1.9)	0.224 (0.004)

Data were presented as mean (standard deviation).

In terms of the correlation across all eight limb segments, the correlation coefficient was found to be higher in children (R = 0.950–0.997, Table 2) as compared to adults (R = 0.783–0.982, Table 3) by DXA. When Azure Kinect was used for evaluation, it shown a perfect correlation among all limb segments (R = 1, Table 4). This suggests that the Azure Kinect Body Tracking SDK assumed fixed limb proportions beforehand.

**Table 2:** Correlation table of DXA measurement in children.

	Lt arm	Rt arm	Lt forearm	Rt forearm	Lt thigh	Rt thigh	Lt leg	Rt leg
Lt arm	1	.995**	.959**	.951**	.960**	.958**	.973**	.969**
Rt arm	.995**	1	.950**	.950**	.961**	.957**	.968**	.963**
Lt forearm	.959**	.950**	1	.981**	.971**	.970**	.963**	.965**
Rt forearm	.951**	.950**	.981**	1	.984**	.985**	.959**	.959**
Lt thigh	.960**	.961**	.971**	.984**	1	.997**	.974**	.972**
Rt thigh	.958**	.957**	.970**	.985**	.997**	1	.968**	.968**
Lt leg	.973**	.968**	.963**	.959**	.974**	.968**	1	.992**
Rt leg	.969**	.963**	.965**	.959**	.972**	.968**	.992**	1

**p < 0.0001.

**Table 3:** Correlation table of DXA measurement in adults.

	Lt arm	Rt arm	Lt forearm	Rt forearm	Lt thigh	Rt thigh	Lt leg	Rt leg
Lt arm	1	.943**	.855**	.882**	.911**	.919**	.828**	.865**
Rt arm	.943**	1	.841**	.871**	.886**	.891**	.783**	.814**
Lt forearm	.855**	.841**	1	.938**	.873**	.878**	.838**	.841**
Rt forearm	.882**	.871**	.938**	1	.902**	.901**	.812**	.853**
Lt thigh	.911**	.886**	.873**	.902**	1	.982**	.833**	.848**
Rt thigh	.919**	.891**	.878**	.901**	.982**	1	.846**	.859**
Lt leg	.828**	.783**	.838**	.812**	.833**	.846**	1	.963**
Rt leg	.865**	.814**	.841**	.853**	.848**	.859**	.963**	1

**p < 0.0001.

**Table 4:** Correlation table of Kinect measurement.

	Lt arm	Rt arm	Lt forearm	Rt forearm	Lt thigh	Rt thigh	Lt leg	Rt leg
Lt arm	1	1.000**	1.000**	1.000**	1.000**	1.000**	1.000**	1.000**
Rt arm	1.000**	1	1.000**	1.000**	1.000**	1.000**	1.000**	1.000**
Lt forearm	1.000**	1.000**	1	1.000**	1.000**	1.000**	1.000**	1.000**
Rt forearm	1.000**	1.000**	1.000**	1	1.000**	1.000**	1.000**	1.000**
Lt thigh	1.000**	1.000**	1.000**	1.000**	1	1.000**	1.000**	1.000**
Rt thigh	1.000**	1.000**	1.000**	1.000**	1.000**	1	1.000**	1.000**
Lt leg	1.000**	1.000**	1.000**	1.000**	1.000**	1.000**	1	1.000**
Rt leg	1.000**	1.000**	1.000**	1.000**	1.000**	1.000**	1.000**	1

**p < 0.0001.

Table 5 presents the results of the correlation and agreement analysis for the eight major limb lengths when comparing the Azure Kinect with DXA method in both adults and children. Overall, measurements in children showed a stronger correlation and agreement with both methods. An analysis of Subgroups comprising both obese and non-obese adults shows similar results (Supplementary Table 2). For the correlation analysis, both methods showed a very strong correlation (R = 0.850–0.918) and exhibited excellent linearity (R^2^ = 0.723–0.843) in the adult group. In the child group, measurements by both tools showed a very strong correlation (R = 0.944–0.985) and almost perfect linearity for linear regression (R^2^ = 0.927–0.971), except for the measurement in the right forearm, which demonstrated an excellent fit (R^2^ = 0.891).

**Table 5:** Correlation and agreement between DXA and Kinect measurements.

	Correlation	R^2^	Regression equation	Agreement		Bland–Altman plot	
				ICC	CCC	Bias	95% CI (%)
Children							
Lt arm (cm)	0.982	0.964	y = − 3.5277 + 1.1477x	0.970	0.968	0.162	− 0.149 to 0.473
Rt arm (cm)	0.972	0.945	y = − 3.2999 + 1.1197x	0.961	0.959	− 0.254	− 0.604 to 0.0957
Lt forearm (cm)	0.964	0.929	y = − 0.3127 + 1.0405x	0.937	0.934	0.536	0.251 to 0.821
Rt forearm (cm)	0.944	0.891	y = − 0.7025 + 1.0430x	0.938	0.935	0.211	− 0.153 to 0.574
Lt thigh (cm)	0.963	0.927	y = − 4.0501 + 1.1259x	0.945	0.944	0.507	− 0.0675 to 1.081
Rt thigh (cm)	0.964	0.929	y = − 4.6627 + 1.1432x	0.945	0.942	0.516	− 0.0701 to 1.102
Lt calf (cm)	0.982	0.964	y = − 3.2358 + 1.0890x	0.977	0.976	− 0.157	− 0.527 to 0.213
Rt calf (cm)	0.985	0.971	y = − 2.2165 + 1.0466x	0.973	0.971	− 0.589	− 0.893 to − 0.284
Adult							
Lt arm (cm)	0.896	0.803	y = 1.9202 + 0.9311x	0.896	0.894	0.078	− 0.096 to 0.252
Rt arm (cm)	0.850	0.723	y = 3.0844 + 0.8751x	0.831	0.829	− 0.317	− 0.529 to − 0.105
Lt forearm (cm)	0.869	0.755	y = − 0.6390 + 1.0426x	0.831	0.828	0.317	0.130 to 0.504
Rt forearm (cm)	0.854	0.729	y = − 1.3106 + 1.0655x	0.829	0.826	0.177	− 0.030 to 0.384
Lt thigh (cm)	0.865	0.749	y = 0.7157 + 0.9904x	0.849	0.847	0.344	0.034 to 0.654
Rt thigh (cm)	0.877	0.770	y = − 0.1961 + 1.0159x	0.854	0.852	0.420	0.119 to 0.720
Lt leg (cm)	0.918	0.843	y = − 1.6648 + 1.0214x	0.835	0.762	− 0.873	− 1.101 to − 0.644
Rt leg (cm)	0.904	0.816	y = − 0.2887 + 0.9688x	0.712	0.708	− 1.455	− 1.696 to − 1.214

Bias in Bland–Altman plot is calculated as (DXA-Kinect)/mean.

In the agreement analysis, it was observed that all limb length measurements obtained through both methods exhibited good agreement (ICC = 0.829–0.896) in adults. However, for the right leg in adults, the agreement was slightly lower but still considered moderate (ICC = 0.712). In contrast, measurements taken from children using both methods demonstrated excellent agreement (ICC = 0.937–0.977).

Concordance Correlation Coefficient (CCC) was used to evaluate the precision and accuracy of measurements obtained from DXA and Azure Kinect methods. In the adult group, it was observed that there was poor agreement between the measurements by the two methods (CCC = 0.708–0.894). Conversely, in the child group, the measurements showed moderate to substantial agreement (CCC = 0.934–0.976).

The Bland–Altman Plot analysis as used to test the difference between DXA and Kinect measures, showing that the measurement bias between the two methods for each major limb segments ranged from − 0.589 to 0.536 cm in children and from − 1.455 and 0.420 cm in adults.

## Discussion

This research explores the application of the Azure Kinect for estimating limb length in healthy subjects, using low-dose X-ray method DXA as the reference method. The length of a limb can be measured using mechanical, anatomic or kinematic axis^13^. In this study, limb lengths by DXA were estimated using mechanical axis because it is more compatible to the Azure Kinect method. This study showed that there is a reliable correlation and agreement in estimating limb length between the two methods across both children and adults. These findings indicate the potential of the Azure Kinect as a valuable tool for anthropometric assessments. However, the Azure Kinect assumes that all limb lengths have the same fixed proportion for each person. This feature might not be suitable for certain studies, such as those aim to assess limb discrepancies or anatomical differences in people.

In clinical settings, the DXA method is commonly used to estimate bone mineral densities and body composition. This study is distinctive for utilizing the DXA method but not conventional X-ray method as a radiographic anthropometric technique. DXA scans and conventional X-ray methods both create images by projecting the 3D structure onto a 2D film, which can introduce measurement errors in body segment lengths. However, the DXA scanner exhibits less magnification and distortion errors along the body’s vertical axis (from head to toe) compared to conventional X-ray methods^12,15^. This is because DXA scan uses a movable C-arm gantry containing an X-ray tube and a linear detector array, which moves in a line-by-line pattern along the vertical axis of the body. The DXA method also benefits from only using the daily background radiation. This results in images that may have less detail but are still adequate for observing bones and joints, making it a more suitable option for healthy subjects. It is worth noting that the whole-body image in a DXA report is not in the correct size and the image needs to be calibrated according to a known dimension before taking measurements^11,12,16^.

In the human body, there is a minor variation in the body proportions among individuals due to age, genetic factors, nutrition, disease and other factors^17–19^. However, we observe that limb length data obtained from Azure Kinect demonstrate consistent limb length proportions for each subject. A potential explanation for the results by Azure Kinect may be attributed to the limitation imposed by pre-existing assumption in the Azure Kinect model. Previous studies^20–22^ have employed a priori constraints on limb length proportions in the 3D human pose estimation. This approach demonstrates commendable generalization capabilities while avoiding error estimation. Hence, caution should be taken when using Kinect sensor for measuring limb length discrepancies and body proportions.

In this study, the DXA method serves as the reference method for Azure Kinect. Thus, it is important to recognize the inherent difference in imaging technologies between DXA and Kinect. The DXA and Azure Kinect methods were obtained with participants in different positions: the DXA scan was conducted with participants in a supine position, whereas the Azure Kinect imaging involved participants in a standing position. Moreover, DXA images constitute a 2D radiographic measurement, whereas Kinect employs a 3D measurement approach. As participants lay on the examination table during a DXA scan, their limbs were aligned parallel to the table, reducing perspective errors. In this study, participants were positioned on the DXA table with their joints fully extended to further prevent errors caused by the rotational displacement of the limbs. Despite the inherent differences between DXA and Azure Kinect methods, the limb length estimates demonstrated compatibility, indicating that Azure Kinect could serve as an alternative tool for limb length measurement.

In this study, both children and adults have similar limb-to-height ratios, measured by either DXA or Azure Kinect methods. In general, the lengths of the arm, forearm, thigh, and leg constitute 16%, 14%, 23%, and 22% of the body’s height, respectively, across both age groups. These findings regarding body proportions align with earlier researches using clinical anthropometric measurement^23,24^. This study also discovered that body proportions were less variations in children compared to adults. This might be because lifestyle differences are more apparent in adults. However, no relevant references were found.

While Azure Kinect shows promise in measuring body limb length, it also has limitations. Firstly, external factors, like environmental lighting and object color, can cause noise in the Azure Kinect readings^25^. To minimize the noise, we conducted our study in a room without windows, ensuring consistent artificial lighting. Participants were also instructed to wear light-colored clothing during the examination. Secondly, viewing angles and position of the object might affect the measurement errors of Kinect sensor^26,27^. To deduce the error, Azure Kinect was placed right in frontal of the participants and as close as possible to the subject. Thirdly, a previous study suggested that being obese might affect the accuracy of 3D mesh reconstruction^5^. However, our findings indicated that obesity does not appear to restrict the Kinect sensor’s ability to track body joint locations. Finally, the Kinect software’s built-in assumptions and tracking algorithms for skeletal tracking may restrict the accurate placement of joint locations, leading to measurement errors^28^. As we lack knowledge about these assumptions and algorithms, we cannot prevent these errors.

In conclusion, Azure Kinect, equipped with its integrated software, can automatically capture joint coordinates without the need for body markers. Our research shows that Azure Kinect yields similar outcomes as DXA, indicating its potential utility as a tool for ergonomic studies. However, the built-in algorithm of Azure Kinect assumes fixed limb proportions for individuals, which may not be ideal for studies focuses on investigating limb discrepancies or anatomical differences.

### Supplementary Information

Supplementary Tables.

## Data Availability

The data that support the findings of this study are available on request from the corresponding author. The data are not publicly available due to privacy or ethical restrictions.
