# Supplementary material for: Performance of Microsoft Azure Kinect DK as a tool for estimating human body segment lengths
Source: Sci Rep. 2024 Jul 9;14:15811. doi: 10.1038/s41598-024-66798-0 (PMC11233572; doi:10.1038/s41598-024-66798-0)
Supplement: Supplementary file 1 — Supplementary Tables. [file 41598_2024_66798_MOESM1_ESM.pdf]

Performance of Microsoft Azure Kinect DK as a tool for estimating human body segment lengths

Shiou-An Wang[1], Ming-Hua Lu[1,2], Ai-Teng Lee[2], Chao-Yu Chen[3,4], Li-Wen Lee[2,5,*]

[1]Department of Computer Science and Information Engineering, Hungkuo Delin University of Technology, New Taipei City 236302, Taiwan

[2]Department of Diagnostic Radiology, Chang Gung Memorial Hospital, Chiayi 613016, Taiwan

[3]Department of Obstetrics and Gynecology, Chang Gung Memorial Hospital, Chiayi 613016, Taiwan

[4]Graduate Institute of Clinical Medical Sciences, College of Medicine, Chang Gung University, Taoyuan 33302, Taiwan

[5]School of Medicine, College of Medicine, Chang Gung University, Taoyuan 33302, Taiwan

Supplementary Table 1. Limb length and corresponding anatomic landmarks by Azure Kinect DK.

Length	Proximal end		Distal end	
	Code	Joint name	Code	Joint name
Lt upper arm	joint [5]	SHOULDER_LEFT	joint [6]	ELBOW_LEFT
Lt forearm	joint [6]	ELBOW_LEFT	joint [7]	WRIST_LEFT
Rt upper arm	joint [12]	SHOULDER_RIGHT	joint [13]	ELBOW_RIGHT
Rt forearm	joint [13]	ELBOW_RIGHT	joint [14]	WRIST_RIGHT
Lt thigh	joint [18]	HIP_LEFT	joint [19]	KNEE_LEFT
Lt leg	joint [19]	KNEE_LEFT	joint [20]	ANKLE_LEFT
Rt thigh	joint [22]	HIP_RIGHT	joint [23]	KNEE_RIGHT
Rt leg	joint [23]	KNEE_RIGHT	joint [24]	ANKLE_RIGHT

Supplementary Table 2. Correlation and agreement between DXA and Kinect measurements in adults with and without obesity.

				Agreement		Bland-Altman plot	
	Correlation	R^2	Regression Equation	ICC	CCC	Bias	95% CI (%)
**BMI ≤ 25 kg/m^2 (n=37)**							
Lt arm (cm)	0.874	0.764	y = 0.3550 + 0.9944 x	0.860	0.857	0.207	-0.029 to 0.442
Rt arm (cm)	0.869	0.755	y = 0.1019 + 0.9907 x	0.860	0.857	-0.147	-0.391 to 0.098
Lt forearm (cm)	0.855	0.730	y = -2.0333 + 1.1091 x	0.794	0.789	0.375	0.131 to 0.619
Rt forearm (cm)	0.849	0.720	y = -3.4563 + 1.1651 x	0.799	0.794	0.236	-0.033 to 0.505
Lt thigh (cm)	0.841	0.707	y = -1.4297 + 1.0498 x	0.804	0.800	0.467	0.050 to 0.884
Rt thigh (cm)	0.859	0.738	y = -2.4823 + 1.0792 x	0.814	0.810	0.533	0.134 to 0.932
Lt calf (cm)	0.882	0.779	y = 0.9965 + 0.9480 x	0.804	0.783	-0.899	-1.198 to -0.560
Rt calf (cm)	0.880	0.774	y = 1.2751 + 0.9286 x	0.814	0.687	-1.352	-1.654 to -1.050
**BMI > 25 kg/m^2 (n=17)**							
Lt arm (cm)	0.960	0.922	y = 0.1211 + 0.9883 x	0.948	0.944	-0.201	-0.368 to -0.034
Rt arm (cm)	0.793	0.629	y = 4.3957 + 0.8194 x	0.683	0.670	-0.688	-1.076 to -0.300
Lt forearm (cm)	0.871	0.758	y = -1.2274 + 1.0611 x	0.847	0.839	0.190	-0.105 to 0.484
Rt forearm (cm)	0.832	0.692	y = -0.1987 + 1.0105 x	0.825	0.816	0.049	-0.284 to 0.382
Lt thigh (cm)	0.901	0.811	y = -0.5099 + 1.0146 x	0.899	0.894	0.075	-0.337 to 0.487
Rt thigh (cm)	0.898	0.807	y = -1.3561 + 1.0382 x	0.890	0.884	0.172	-0.256 to 0.601
Lt calf (cm)	0.948	0.899	y = -8.7855 + 1.2083 x	0.838	0.830	-0.816	-1.182 to -0.450

Rt calf (cm)	0.937	0.878	y = -10.5172 + 1.2286 x	0.641	0.627	-1.673	-2.095 to -1.262

Note: Bias in Bland-Altman plot is calculated as (DXA-Kinect)/mean.
